# Metabolic and Hormonal Changes in Pediatric Burn Patients: Mechanisms, Evidence, and Care Strategies

**DOI:** 10.3390/ebj6020017

**Published:** 2025-04-07

**Authors:** Gloria Pelizzo, Valeria Calcaterra, Michela Marinaro, Paola Baldassarre, Carlotta Paola Maria Canonica, Gianvincenzo Zuccotti

**Affiliations:** 1 Department of Biomedical and Clinical Science, University of Milan, 20157 Milan, Italy; gloriapelizzo@gmail.com (G.P.); gianvincenzo.zuccotti@unimi.it (G.Z.); 2 Pediatric Surgery Department, Buzzi Children’s Hospital, 20154 Milan, Italy; michelamarinaro09@gmail.com (M.M.); carlotta.canonica@unimi.it (C.P.M.C.); 3 Pediatrics and Adolescentology Unit, Department of Internal Medicine, University of Pavia, 27100 Pavia, Italy; 4 Pediatric Department, Buzzi Children’s Hospital, 20154 Milan, Italy; paola.baldassarre@unimi.it

**Keywords:** burns, pediatrics, children, metabolic changes, hormonal changes, care strategies, pharmacological interventions, pediatric surgery

## Abstract

Background: Burn injuries constitute a significant global health challenge, especially in pediatric populations, where they are a leading cause of morbidity and mortality. Pediatric burns require particular attention due to their unique pathophysiology, long-term consequences on growth and development, and psychological impacts. Methods: We propose a comprehensive review of recent advancements in understanding the key aspects of hormonal and metabolic changes in burned children, aiming to guide therapeutic interventions, improve outcomes, and reduce the global burden of these injuries. Results: Effective management of the physiological stress response in pediatric burn patients necessitates a multidisciplinary approach integrating medical, nutritional, and rehabilitative strategies. Timely nutritional support and individualized plans preserve muscle mass, promote wound healing, and reduce complications and organ dysfunction risk. Advances in pharmacological interventions, such as beta-blockers, anabolic agents, and hormonal treatment, offer promising pathways to improve recovery and mitigate long-term complications. Early mobilization and physiotherapy are essential for preventing complications of prolonged immobility, including muscle wasting, joint contractures, and functional decline; their effectiveness is closely tied to advancements in minimally invasive procedures, regenerative medicine, and reconstructive techniques, particularly for pediatric patients. Conclusions: While current strategies have significantly improved survival and outcomes for pediatric burn patients, ongoing research is critical to refine these new care strategies.

## 1. Introduction

Burn injuries constitute a significant global health challenge, especially in pediatric populations, where they are a leading cause of morbidity and mortality [1,2]. These injuries, defined as tissue damage caused by thermal, chemical, electrical, or radiation exposure, are complex and can affect the skin as well as deeper structures, such as muscles, nerves, and bones in severe cases [1].

Pediatric burns require particular attention due to their unique pathophysiology, long-term consequences on growth and development, and psychological impacts. Globally, burns among children account for a substantial proportion of emergency department visits and hospital admissions, with the highest incidence reported in low and middle-income countries. Among children under the age of 5, scalds from hot liquids or steam are the most common cause, while flame burns are more prevalent in adolescents. Epidemiological studies indicate that boys are slightly more affected than girls, and children under five years of age are at the greatest risk due to their thinner skin and increased exposure to household hazards [2,3].

Burns are categorized based on depth (ranging from superficial to full thickness), extent (measured by the Total Body Surface Area (TBSA) affected), and causative agent, including thermal, electrical, chemical, and radiation burns, each with distinct clinical features and treatment considerations [1,2,3,4,5]. The severity of burns is stratified into minor, moderate, and severe categories, determined by factors such as depth, TBSA, patient age, and the presence of inhalation injury or comorbidities [1,2,3,4,5]. Specifically, in pediatric classification, severe burns are defined as follows: burns covering more than 10% of the TBSA in children under 10 years old, or more than 20% TBSA in older children (over 10 years old); burns that exceed 5% full thickness, which requires surgical excision and grafting; high-voltage electrical burns, which can lead to deep tissue damage and systemic complications; and significant burns affecting the face, eyes, ears, joints, or genitalia, as these areas are essential for both function and appearance [6,7].

Severe burns, particularly those exceeding 10% TBSA in young children, pose systemic risks such as hypovolemic shock, hypermetabolic response, and multi-organ dysfunction due to the higher surface-to-body size, which are often life-threatening [1,4,5]. Current therapeutic strategies for pediatric burns emphasize both acute management and long-term rehabilitation. Advanced techniques, including skin grafting and tissue-engineered dermal substitutes, are essential for managing extensive burns. Innovations in wound coverage, such as biosynthetic dressings and cultured epithelial autografts, improve outcomes by reducing scarring and enhancing healing rates [8].

Emerging therapies target the systemic effects of severe burns, exploring metabolic modulators and immunomodulatory agents to mitigate hypermetabolism and inflammation. Future directions include regenerative medicine, 3D bioprinting for customized grafts, and gene therapy to optimize tissue repair and functional recovery. Advances in telemedicine and artificial intelligence are also expected to revolutionize burn care by improving early diagnosis, triage, and personalized treatment planning [1,8].

We propose a comprehensive review of recent advancements in understanding the key aspects of hormonal and metabolic changes in children with major burn injuries, aiming to guide therapeutic interventions, improve outcomes, and reduce the global burden of these injuries.

## 2. Methods

We conducted a narrative review to summarize the literature on hormonal and metabolic changes in burn injuries in pediatric patients and comprehensive therapeutic options. To ensure relevance, specific inclusion criteria were applied: only articles published in English that included meta-analyses, clinical trials, original articles, and reviews from the past twenty years were included. Case reports and series were not considered. The research utilized electronic databases such as PubMed and the Web of Science. The search incorporated keywords that included burns OR burn injuries OR major burns OR severe burns AND stress OR stress response OR physiological response AND metabolic changes OR dysmetabolism AND hormonal changes OR hormones AND nutrition OR nutritional support AND children OR pediatric. Initially, the authors reviewed a total of 766 papers, refining our selection by screening abstracts (n = 256). Ultimately, 75 articles met the inclusion criteria. Additionally, the reference lists of all relevant manuscripts were examined to identify further pertinent studies. AI-assisted tools were used for the revision of certain grammatical aspects.

**Figure 1:**
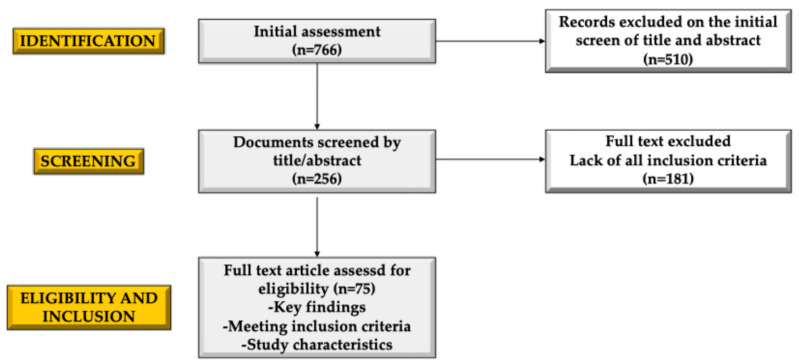
The flowchart of the selection of the manuscript.

A flowchart illustrating the selection process of the manuscript is presented in Figure 1.

## 3. Physiological Response to Burns

**Figure 2:**
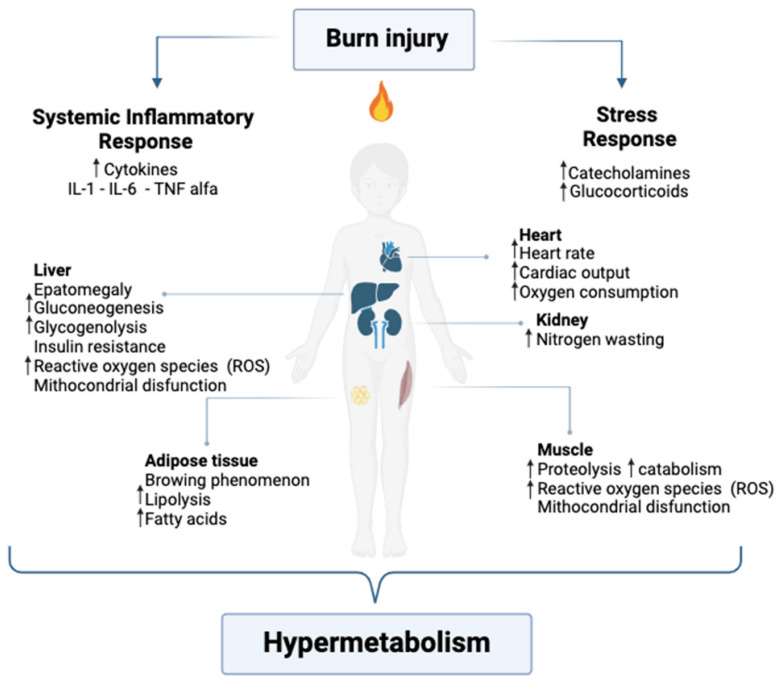
Hypermetabolic state in pediatric burn injury (↑ increased).

Severe burn injuries elicit a complex physiological stress response involving both localized and systemic effects, which profoundly impact recovery trajectories and long-term outcomes. This response is characterized by hypermetabolism, neuroendocrine activation, and a prolonged inflammatory cascade, often persisting well beyond the acute phase of injury [9,10] (Figure 2).

In children, these responses are particularly pronounced and intricate due to their unique metabolic and endocrine characteristics in response to the relationship between BSA involvement, body mass, and their developing physiological systems, which are highly sensitive to changes in metabolic demands and environmental stressors.

The clinical presentation, physiological response, and therapeutic needs of burn injuries vary by age group.

Infants and young children (0–5 years) are most vulnerable to scald burns due to thin skin, limited mobility, and curiosity. Their high TBSA-to-weight ratio increases the risk of burn shock and fluid loss, requiring aggressive fluid resuscitation. Immature immune systems elevate the risk of infection and sepsis, while facial burns can lead to airway swelling. Treatment prioritizes fluid replacement, pain management, wound care, early physiotherapy, and nutritional support to prevent contractures [11,12].

Children (6–12 years) more frequently experience scald and flame burns from cooking accidents, fireworks, or campfires. Their skin is more resistant but still susceptible to deep burns. Smoke inhalation is a risk, and scarring can cause emotional distress. Treatment includes fluid resuscitation, pain management, early physical therapy to preserve mobility, and psychological support for coping with visible scars [1,13,14]. Even in this age group, burns on the face, particularly around the mouth and nose, can lead to rapid airway swelling, which may become a life-threatening situation [1,13,14].

Adolescents (>12 years) face flame, chemical, and electrical burns, often linked to risk-taking behaviors or workplace exposure. Their skin resembles that of adults, making them less prone to rapid fluid loss, but they have a higher risk of hypertrophic scars and contractures. The psychological distress related to body image and social impact is significant, requiring both medical and emotional support [14].

As a result, burn injuries represent one of the most demanding traumatic conditions, requiring an intense multidisciplinary approach to sustain recovery, preserve energy reserves, and support the healing process.

### 3.1. Local Response to Burn Injury

Locally, burn injuries initiate dynamic, interdependent processes to limit tissue damage and promote repair [1,9].

The inflammatory response is activated immediately post-injury and may persist for months. Affected tissues are classified into three distinct zones: the zone of coagulation, where thermal damage causes irreversible cellular necrosis; the zone of stasis, marked by reduced perfusion and salvageable tissue that is highly susceptible to necrosis without timely intervention; and the zone of hyperemia, characterized by increased blood flow due to inflammatory vasodilation [1].

Burn injuries induce extensive vascular damage and capillary leakage, driven by endothelial cell activation. Necrotic cells release damage-associated molecular patterns, recruiting immune cells such as neutrophils and macrophages. Neutrophils generate reactive oxygen species (ROS) and proteolytic enzymes to clear debris, but their dysregulated activity can amplify tissue damage. Macrophages exhibit functional plasticity, transitioning between pro-inflammatory (M1) and anti-inflammatory (M2) phenotypes, thereby playing a pivotal role in the shift from inflammation to tissue repair [15]. Key pro-inflammatory cytokines, including interleukin (IL)-1, IL-6, IL-8, and tumor necrosis factor-alpha (TNF-α), further amplify the inflammatory response. These effects in pediatric patients are magnified due to their distinct metabolic demands and ongoing developmental processes, perpetuating a self-reinforcing cycle of inflammation, hypermetabolism, and immune dysfunction. Cytokines mediate vasodilation and increase vascular permeability, leading to edema that can exacerbate ischemia within the zone of stasis. Concurrent activation of the complement system and coagulation pathways promotes a pro-thrombotic state, worsening perfusion deficits [16].

Burn injuries also disrupt keratinocytes, fibroblasts, and endothelial cells, compromising skin integrity and its regenerative potential. Growth factors, including vascular endothelial growth factor (VEGF) and fibroblast growth factors (FGFs), activate keratinocytes and fibroblasts, initiating the proliferative phase of wound healing. However, extensive necrosis in severe burns delays re-epithelialization and granulation tissue formation. Additionally, the inflammatory microenvironment upregulates matrix metalloproteinases (MMPs), which degrade the extracellular matrix and impair structural repair. In the zone of stasis, ischemia caused by microvascular injury and edema induces tissue hypoxia, which, if unresolved, progresses to irreversible necrosis. Paradoxically, reperfusion injury is driven by an influx of ROS and inflammatory mediators, further exacerbating local damage [1,16]. Burn wounds lack the compartmentalized inflammatory response typical of other injuries due to the destruction of the skin’s structural and immunological barriers. This exposes deeper tissues to microbial invasion, heightening the risk of infection. Additionally, the loss of skin integrity compromises thermoregulation, increases transepidermal water loss, and predisposes patients to secondary complications [1,17].

The local response to burn injury represents a delicate balance between damage limitation and tissue repair. In pediatric patients, thinner skin and an exaggerated inflammatory response amplify tissue damage, necessitating prompt intervention to preserve viable tissue in the zone of stasis and facilitate the reparative phase [18].

### 3.2. Metabolic Changes

#### 3.2.1. Hypermetabolic and Inflammatory Response

In response to a major burn, the pediatric body initiates a hypermetabolic response by a significant increase in systemic oxygen consumption and ATP expenditure, with resting energy expenditure (REE) rising by up to 200%, substantially higher than in adults [19]. Additionally, the basal metabolic rate remains elevated by 110% for over a year [20]. This sustained elevation in REE reflects the energy-intensive demands of tissue repair, immune activation, and thermoregulation, which are critical for supporting the child’s recovery.

The extent of the hypermetabolic response correlates with the TBSA burned, with larger burns triggering more pronounced increases in REE [21]. This prolonged hypermetabolism, compounded by growth and nutritional needs, can deplete energy stores, cause muscle wasting, and hinder long-term development [22,23,24]. Thus, in pediatric burn patients of all ages, particularly those under 5 years old, maintaining an adequate energy supply is critical due to their higher basal metabolic rate and limited energy reserves compared to adults [17].

Suman et al. [25] highlighted that if the heightened REE is not supported with aggressive nutritional support, it can rapidly lead to the depletion of lean body mass and fat stores, exacerbating muscle wasting and impairing immune function. This loss of lean body mass further perpetuates the hypermetabolic state, creating a vicious cycle that delays recovery and increases the risk of complications, including wound infections and sepsis [17,26].

The systemic catabolic state induced by severe burns precipitates immune dysfunction. The inflammatory response to burns causes profound alterations in innate and adaptive immune systems. In children, innate immunity becomes hyperactivated [18], with increased pro-inflammatory signaling and acute-phase protein synthesis, including elevated C-reactive protein (CRP) levels. In contrast, adaptive immunity is suppressed, characterized by impaired T-lymphocyte activity and altered cytokine production. This immune dysregulation heightens susceptibility to infections and sepsis, which remain the leading causes of mortality in burn patients [18].

The hypermetabolism is driven by sustained elevations in catecholamines, cortisol, glucagon, and dopamine, alongside increased levels of IL-1, IL-6, tumor necrosis factor-alpha (TNF-α), platelet-activating factor (PAF), complement activation, and enhanced production of ROS [27]. The stress response evolves in two distinct phases: the early “ebb” phase and the subsequent “flow” phase. The ebb phase begins immediately after injury, lasting approximately three days, and is characterized by reduced cardiac output, decreased oxygen consumption, and transient hyperglycemia. As the systemic inflammatory response intensifies, it transitions to the flow phase, a hypermetabolic state marked by heightened metabolism and circulatory alterations that can persist indefinitely, with substantial physiological repercussions [2].

This stress response also affects multiple organ systems. The cardiovascular system undergoes hyperdynamic changes, including increased cardiac output, tachycardia, and elevated myocardial oxygen consumption, which may lead to myocardial dysfunction. Cardiac function is notably affected, with increased heart rate and cardiac output persisting post-burn. The liver experiences hypertrophy and heightened metabolic activity to sustain acute-phase protein synthesis, but this is frequently accompanied by hepatic dysfunction, manifested as elevated transaminases and impaired glucose metabolism [20,28].

Following the hypermetabolic response described, hormonal imbalances also become evident, further exacerbating metabolic inefficiencies [1,4,10]. A detailed discussion of hormonal changes is provided in the following paragraphs.

#### 3.2.2. Catabolism and Muscle Degradation

Protein catabolism is a critical component of the metabolic response to burns, especially in pediatric patients, where it can lead to a considerable loss of lean body mass. Due to a hypermetabolic and hypercatabolic state, severely burned children may lose as much as 20% of their muscle mass within the first few weeks following injury.

This significant muscle loss primarily arises from the body’s heightened demand for amino acids to facilitate gluconeogenesis, promote wound healing, and support immune function [17].

Children, unlike adults, possess relatively limited muscle reserves, rendering them more vulnerable to the adverse effects of muscle degradation [29]. In addition, the ongoing hypermetabolic response further compounds muscle mass loss by increasing protein turnover while decreasing the synthesis of new muscle proteins [25].

Skeletal muscle plays a central role in the body’s response to burns, acting as a primary reservoir of amino acids, such as alanine, to support wound healing, gluconeogenesis, and acute-phase protein synthesis. However, the balance between muscle protein synthesis and degradation is significantly disrupted, with proteolysis exceeding synthesis, leading to muscle wasting and increased morbidity and mortality. The net catabolic state persists for over a year in pediatric patients, with lean body mass reductions observed for 2–3 years post-injury. Proteolysis is driven by the activation of the ubiquitin-proteasome system, a proteolytic pathway that degrades muscle proteins [15,30]. This process is further exacerbated by elevated systemic levels of stress hormones and pro-inflammatory cytokines, including IL-6 and TNF-α, which upregulate muscle proteolysis and interfere with anabolic signaling pathways such as insulin signaling. Two distinct phases of disruption may be distinguished. The acute resorption phase occurs within the first two weeks post-injury, driven by elevated cytokines such as IL-1β and IL-6. The subsequent phase is characterized by reduced bone formation, leading to an adynamic bone state with low turnover. Pediatric burn patients may lose up to 7% of lumbar spine bone mass within three weeks, with total body bone mass decreasing by 3% at six months post-burn. This condition contributes to growth delays, bone loss, and further lean mass depletion, perpetuated by elevated glucocorticoid secretion, vitamin D deficiency, and calcium loss. Hypoparathyroidism may also exacerbate these effects [15,30,31,32]. Total body bone mineral content decreases by 3%, with cortical bone recovery within 18–24 months, while trabecular bone mass remains compromised. Consequently, children with severe burns are at increased risk for reduced peak bone mass, future osteoporosis, and fracture rates [30,33]. Long-term muscle loss can also hinder growth and skeletal development, contributing to growth retardation in children with severe burns [32,34].

Exercise and rehabilitation are critical for recovery. Structured resistance training has been demonstrated to improve muscle strength and functional mobility in children with severe burns [35]. In pediatric burn patients, a comprehensive approach combining nutritional support, pharmacological therapy, and physical rehabilitation offers the best chance for long-term recovery.

#### 3.2.3. Negative Nitrogen Balance

Burn injuries result in a negative nitrogen balance, where the breakdown of proteins exceeds their synthesis. This imbalance is a key feature of the hypercatabolic state that follows severe burns, resulting in significant nitrogen loss in urine, which indicates extensive muscle protein degradation. Such nitrogen loss impairs a child’s ability to rebuild tissues and recover lean body mass. Children are particularly at risk due to their higher protein turnover rates and lower muscle reserves compared to adults, which increases the likelihood of delayed wound healing, weakened immune function, and growth retardation [36].

Urinary nitrogen excretion can remain elevated for weeks to months after the injury, often correlating with the TBSA affected. This prolonged nitrogen imbalance contributes to ongoing muscle wasting and functional impairment, extending the recovery and rehabilitation process for pediatric patients [10].

The most effective approach to managing nitrogen loss in severely burned children involves a combination of high-protein nutrition, pharmacological therapies, and early physical rehabilitation. Together, these strategies help mitigate protein degradation, preserve lean body mass, and support recovery in this vulnerable population.

#### 3.2.4. Lipolysis and Oxidative Stress

In response to the increased energy demands following severe burns, lipolysis is significantly accelerated, resulting in the release of free fatty acids (FFAs) as an alternative energy source [37]. This contributes to hepatomegaly, steatosis, and impaired fat oxidation, perpetuating hypermetabolism and muscle catabolism. A phenomenon known as adipose tissue browning, characterized by white adipocytes adopting a brown, thermogenic phenotype mediated by UCP-1 and possibly IL-6, exacerbates hypermetabolism. This process promotes lipotoxicity, insulin resistance, and futile triglyceride cycling, further compounding the systemic metabolic disruption [30,38,39].

This mechanism is crucial for meeting the heightened metabolic requirements during the acute phase of injury. However, prolonged lipolysis can lead to the overproduction of ROS, contributing to oxidative stress. Pediatric burn patients are particularly vulnerable to these effects due to their limited antioxidant defenses and higher metabolic demands relative to their body size.

Oxidative stress has been linked to immune dysfunction, delayed wound healing, and an increased risk of infections. Excessive ROS production damages cellular membranes, proteins, and DNA, further impairing tissue repair and prolonging recovery [37]. The immune dysfunction caused by oxidative stress heightens the susceptibility to opportunistic infections, which remain a leading cause of morbidity and mortality in pediatric burn patients [40].

Recent evidence highlights mitochondrial dysfunction in skeletal muscle post-burn, with the uncoupling of oxidative phosphorylation leading to increased heat generation and hypermetabolism. This process elevates ROS production, impairing mitochondrial efficiency and contributing to muscle degradation. Elevated uncoupling proteins (UCP-2 and UCP-3) further reduce ATP production, while mitochondrial stress responses fail to counteract damage effectively [41]. Additionally, systemic inflammation, mediated by factors such as inducible nitric oxide synthase (iNOS) and NF-κB, exacerbates insulin resistance and muscle atrophy [30,42].

In summary, while accelerated lipolysis provides a vital energy source during the hypermetabolic phase following burns, the associated oxidative stress presents significant challenges in pediatric burn care. A combined approach that includes nutritional support, antioxidant supplementation, and strategies to reduce metabolic stress may help optimize recovery and improve long-term outcomes in this vulnerable population.

### 3.3. Hormonal Changes

#### 3.3.1. Catecholamines and Sympathetic Nervous System Activation

Catecholamine release is one of the primary responses to burn trauma, increasing up to tenfold in severely burned children [17]. Catecholamines, such as epinephrine and norepinephrine, stimulate an increased heart rate, cardiac output, and metabolic rate by activating the sympathetic nervous system (SNS). In pediatric patients, the smaller body surface area-to-weight ratio and reduced glycogen stores amplify the systemic effects of this response compared to adults. These hormones promote glycogenolysis and lipolysis, which release glucose and fatty acids to meet the heightened energy demands associated with repair mechanisms [37,43,44].

The sustained release of catecholamines can have several negative effects: prolonged exposure to high levels of catecholamines can lead to increased oxidative stress, impaired immune function, and a catabolic state that accelerates muscle wasting. This issue is particularly concerning in pediatric patients, as muscle loss can significantly affect their growth and development, resulting in delayed recovery and longer rehabilitation needs. Furthermore, the combination of heightened sympathetic nervous system (SNS) activation and elevated cortisol levels may further suppress the regenerative capacity of pediatric tissues, complicating the healing process [45,46,47].

Hypercatecholaminemia in pediatric burn patients has been found to persist for months following the initial injury, with some studies reporting sustained SNS hyperactivity for up to 12 months post-burn [23].

This prolonged activation of the SNS has been linked to several negative outcomes, including reduced bone mineral density, increased insulin resistance, and delayed wound healing. These findings highlight the importance of implementing targeted interventions to address and mitigate the long-term effects of sustained SNS activation [4,48].

#### 3.3.2. Cortisol and Hypothalamic–Pituitary–Adrenal (HPA) Axis

Cortisol plays a vital role in the body’s response to stress. After a burn injury, cortisol levels peak within a few days and remain elevated. This increase in cortisol promotes gluconeogenesis and protein breakdown, supplying the body with energy substrates [37].

However, chronic elevation of cortisol, known as hypercortisolemia, can have several harmful effects, especially in children: prolonged high cortisol levels contribute to hyperglycemia, insulin resistance, and immunosuppression, and hyperglycemia can heighten the risk of infections and sepsis, which are significant complications for burn patients [49,50,51].

Furthermore, extended exposure to cortisol dampens the immune response, reducing lymphocyte function and increasing vulnerability to opportunistic infections [52,53].

Cortisol also plays a direct role in delaying tissue repair processes. Its inhibitory effects on collagen synthesis can impair wound healing and increase the risk of hypertrophic scarring, which is particularly common in pediatric patients due to their active fibroblastic response. Additionally, excessive cortisol disrupts bone metabolism by reducing calcium absorption and increasing bone resorption. When combined with prolonged immobility, this elevates the risk of osteoporosis and stunted growth in children [54,55].

The long-term effects of prolonged activation of the hypothalamic–pituitary–adrenal (HPA) axis highlight the importance of early interventions to manage cortisol levels. Strategies such as nutritional optimization, physical therapy, and pharmacological treatments aim to reduce the adverse effects of hypercortisolemia while supporting the critical energy demands of recovery in pediatric burn patients [4,8].

#### 3.3.3. Thyroid Hormones and Euthyroid Sick Syndrome

Severe burn injuries can significantly impact thyroid hormone levels, often resulting in a condition known as euthyroid sick syndrome (ESS). This condition is characterized by decreased levels of triiodothyronine (T3) and thyroxine (T4) while thyroid-stimulating hormone (TSH) levels remain normal or slightly low [56,57].

In pediatric burn patients, ESS is particularly concerning due to children’s higher metabolic demands and the crucial role of thyroid hormones in growth, development, and metabolic balance [58,59].

Reduced T3 and T4 levels also can impair protein synthesis and delay wound healing, both essential for recovery in growing children [60].

Additionally, thyroid dysfunction can worsen thermoregulatory challenges. Pediatric burn patients are already more susceptible to excessive heat loss due to their larger surface area-to-mass ratio and compromised skin barrier, and this necessitates increased energy expenditure to maintain core body temperature. The suppression of thyroid hormones exacerbates this imbalance, leading to prolonged hypothermia and energy deficiency [61,62,63]. Closely monitoring thyroid function and adopting a balanced treatment approach are essential to avoid disrupting the body’s natural hormonal adaptations while effectively managing the long-term effects of ESS [62,63,64].

#### 3.3.4. Insulin and Insulin Resistance

Hyperglycemia and insulin resistance are common in pediatric burn patients due to the combined effects of cortisol, catecholamines, and the release of pro-inflammatory cytokines. These factors contribute to a hypermetabolic state that increases hepatic glucose production while impairing glucose uptake in peripheral tissues, such as muscle and adipose tissue [65,66].

Persistent hyperglycemia is linked to negative clinical outcomes, including increased infection rates, delayed wound healing, and impaired immune function—factors that can prolong hospitalization and recovery time. Additionally, prolonged hyperglycemia intensifies the catabolic state, speeding up the loss of muscle mass and hindering the recovery process [65,67].

Insulin resistance is evident within the first week post-burn and can persist for up to three years. Maria Chondronikola et al. [68] identified significant predictors factors that are associated with long-term insulin resistance in survivors of pediatric burn injury including burn size, time post-burn, age, lean mass, and adiposity. The study demonstrated that 24 to 36 months after burn injury, a significant proportion of pediatric burn survivors have glucose abnormalities, which should be investigated after burn injury. Data were confirmed by Gauglitz et al. [69] where glucose levels were significantly elevated at 6 months post-burn (*p* < 0.05), associated with significant increases in serum C-peptide and insulin, which remained significantly elevated at 36 months compared with non-burned children (*p* < 0.05). Cross-talk between tissues—including the liver, adipose tissue, and skeletal muscle—further amplifies metabolic dysfunction.

#### 3.3.5. Growth Hormone (GH) and IGF-1 Dysregulation

Growth hormone (GH) and insulin-like growth factor-1 (IGF-1) are essential for growth and tissue repair. After a burn injury, GH levels often increase; however, this rise is typically associated with a condition known as “GH resistance”, where IGF-1 levels remain low. This leads to impaired protein synthesis, reduced muscle recovery, and delayed tissue repair [17].

Pediatric patients are particularly susceptible to these effects, as IGF-1 is essential for linear growth, skeletal development, and muscle regeneration during critical phases of childhood development. The disruption of the GH/IGF-1 axis can lead to growth retardation and prolonged rehabilitation in children with severe burns. Herndon et al. [21,70,71,72] showed that low IGF-1 levels exacerbate the catabolic state, resulting in increased muscle wasting and impaired wound healing. Furthermore, insufficient IGF-1 signaling negatively impacts bone growth, heightening the risk of long-term growth stunting and skeletal abnormalities, which can persist even after the acute recovery phase [32,36].

## 4. Long-Term Consequences for Growth and Development

The hypermetabolic and hypercatabolic responses in children with burn injuries can lead to significant long-term consequences for their growth and development.

These consequences include reduced bone mineral density (BMD) and an increased risk of osteoporosis later in life. Prolonged metabolic stress disrupts the balance between bone formation and resorption, resulting in low bone density that may persist for years after the injury. This is particularly concerning during childhood, a critical period for accumulating peak bone mass [34,73].

The risk of skeletal complications is further exacerbated by immobilization, which is common during burn recovery and accelerates bone resorption and muscle wasting. Prolonged inflammation and elevated cytokine levels also impair the activity of osteoblasts, contributing to reduced BMD [10,32].

Stunted growth is another well-documented outcome, as burn injuries impair the GH/ IGF-1 axis, leading to GH resistance and reduced IGF-1 levels. This hormonal dysregulation, combined with protein catabolism and nutrient deficiencies, delays linear growth and can cause significant height deficits if left untreated [4,25].

Few studies have followed children beyond the acute recovery phase to evaluate growth, bone health, and metabolic function into adolescence and adulthood. More longitudinal research is needed to identify effective therapies and develop comprehensive care strategies that address both the acute and chronic consequences of severe burns in children [1,16,74,75]. In Table 1, key scientific studies on burn patients are reported.

## 5. Pediatric Management of the Stress Response to Burn Trauma

Burns are a traumatic event with a significant impact on pediatric patients. Due to the increased ratio between TBSA and body mass, children are at immediate risk of triggering a cascade of inflammatory, metabolic, and hormonal changes that lead to irreversible damage. Proper management reduces the stress response cascade, especially in large lesions and in small patients who physiologically present thinner skin [78].

### 5.1. Environmental Stewardship

A specialized environment is essential for the effective management of pediatric burns. From the pre-hospital phase through hospitalization and follow-up care, it is crucial to have a dedicated team with expertise in pediatric burn management. Comprehensive pain and stress management should begin immediately following the injury to prevent a cascade of physiological and psychological responses to trauma [2].

It is well established that the inflammatory process and wound healing are closely linked to pain due to the cascade of mediators released both in the short and long term [78].

Burn pain is a spectrum of moments that accounts for acute, breakthrough, procedural, and background pain. Pain management is fundamental to achieving the correct healing process and preventing long-term psychological and behavioral sequelae [78].

Effective pain management should be addressed through dedicated protocols and innovative techniques to minimize discomfort and improve recovery [79,80].

Brown et al. [79] experimented with the use of Ditto^®^, an electronic medical device that aids children to distract and manage procedural pain. Their RCT showed a faster re-epithelization rate in the intervention group and a lower pain score reported compared to standard treatment. Moreover, Chester et al. investigated hypnosis through an RCT, demonstrating the efficacy of this approach tailored and personalized to children’s needs [81]. The evaluation of combined treatments (such as opioids, NSAIDs, and gabapentinoids [82]) can be extremely useful for exploring new therapeutic possibilities for neuropathic pain management in adolescents.

In addition to pain, anxiety and stress-related disorders must also be considered in the care of pediatric burn patients [83]. Scars can lead to social distress, making psychological support an important aspect of long-term care [1,13].

Psychological support is crucial, as many adolescents experience depression, anxiety, or post-traumatic stress disorder after suffering severe burns. Additionally, adolescents may be resistant to rehabilitation measures, such as wearing pressure garments or attending therapy, often due to feelings of embarrassment. Therefore, additional motivation and counseling may be necessary to encourage adherence to treatment [12,84,85,86].

Follow-up care should be tailored to the pediatric burn population to prevent permanent sequelae that can lead to social and psychological disorders over time [87].

### 5.2. Early Wound Excision and Closure

It is well established that the timing of wound incision is correlated with both hospital length of stay and short- and long-term outcomes, such as infection rate and metabolic complications [88,89,90]. Partial- and full-thickness burns should be treated promptly to remove necrotic tissue and facilitate skin regeneration; if not feasible, early grafting should be considered. Deep debridement creates a favorable environment for skin regeneration, helps prevent infections, and facilitates the overall healing process. Early wound excision also prevents the hypermetabolic response associated with severe burns, thereby reducing the physiological parameters linked to stress syndrome [91,92].

Early wound excision has been shown to improve the stress response in pediatric burn patients by reducing the establishment of inflammatory syndrome and modulating the acute phase response to major stress. The management of pediatric burns has also evolved. In the case of severe burns, or mixed-depth lesions, which are the majority of thermal burns in children, the use of enzymatic debridement (Nexobrid^®^) has grown in popularity in the pediatric age over traditional surgery (necrosectomy procedures) [93]. Chemical debridement has the great advantage of identifying the selectivity of the deep areas and preserving the vital dermis, avoiding unnecessary skin grafting and improving the outcome. The main advantage of using this therapy is the number of subsequent reconstructive procedures required due to scar contractures, which is reduced compared to children who had traditional necrosectomy [94].

Furthermore, prompt wound treatment helps prevent the onset of infection and reduces the risk of subsequent blood loss [95,96].

The literature emphasizes the positive impact of early wound excision in the pediatric population, particularly in the context of major burns [94,95,96]. Nevertheless, as most burns in small children are caused by hot liquids, it is crucial to address the varying degrees of lesions within the same areas as soon as possible. Prompt wound treatment reduces the progression of the deep lesions and the risk of subsequent further debridement to promote the healing process.

In addition to wound excision and debridement, skin grafting is the standard treatment for burn injuries. Various techniques have been investigated in the pediatric population, with the scalp being considered an excellent viable donor site for full-thickness lesions. Other options, such as human skin allografts and synthetic grafts, have also been explored as bridge therapies before definitive skin grafting [97,98,99] in severe conditions.

Dermal substitute application, composed of bio-engineered materials or biomimetic constructions of collagen, elastin, and synthetic polymers, should be considered the treatment of choice for pediatric partial-thickness burns, the most common condition in burned children [100]. They promote tissue and facilitate cell migration, wound healing, and regeneration, resulting in a shorter hospital stay, a lower need for escharotomy and grafting, and a lower need for long-term reintervention.

The more recent data from the literature support Negative Pressure Wound Therapy (NPWT) in pediatric ages [101]. This technique has been introduced in clinical practice even in infants (2-month-old patients) with deep second- or third-degree burns of different causes (thermal, chemical, electrical, and frostbite). Even though there is no consensus on the indication, different authors suggest that edema evacuation from the extravascular space decompresses small vessels, increases local blood supply, improves microcirculation and angiogenesis, and increases the viability of the borderline tissues. Moreover, patient mobilization is allowed immediately after the grafting procedure, and a reduction in painful dressing changes, compared to conventional dressing, has been documented.

It is also important to emphasize that burn injuries in critical areas of the body present unique challenges because they significantly affect both physical function and psychological well-being. The complex anatomy and vital functions of the face, hands, feet, and genitalia mean that burns in these regions require specialized treatment and rehabilitation approaches. This targeted care aims to minimize long-term complications and enhance patient outcomes. These burns carry a high risk of complications, including functional impairment, contractures, disfigurement, and psychological distress. Early intervention through multidisciplinary care—incorporating surgical management, rehabilitation, and psychosocial support—is essential to optimize recovery and prevent long-term disabilities [102].

Burns to the face are particularly concerning because they pose a risk of airway compromise, corneal injury, and permanent scarring. The high vascularity of facial tissues leads to rapid swelling, which can obstruct the airway, especially in burns affecting the nose, mouth, and throat. Inhalation injuries may also occur alongside facial burns, necessitating early intubation to secure the airway and prevent respiratory distress [102,103].

In general, any burns of the eyes or eyelids require urgent ophthalmologic consultation. To protect the eyes, lubricating ointments and moisture chambers are used. Beyond acute management, long-term interventions focus on minimizing scarring and restoring facial symmetry. Early excision and skin grafting, along with laser therapy and pressure therapy, help improve cosmetic outcomes and reduce hypertrophic scar formation. Given the psychosocial impact of facial burns, particularly in older children and adolescents, psychological support and reconstructive surgery may be required to aid emotional recovery and social reintegration [104,105].

Burns to the hands are among the most functionally significant injuries in children, as hands are essential for fine motor skills and self-care. The thin skin on the hands, along with the superficial position of tendons, nerves, and joints, makes them highly susceptible to contractures and loss of function if not treated promptly. To prevent joint stiffness and deformities, immediate splinting and positioning of the hands in a functional posture are crucial. Early physical and occupational therapy is vital to maintain range of motion and prevent long-term disability [106]. Without early intervention and ongoing rehabilitation, even minor burns to the hands can lead to significant impacts on dexterity and daily activities, potentially resulting in lifelong functional impairments [102].

Burns on the feet present unique challenges due to their crucial role in weight-bearing and mobility. Scarring and contractures in this area can lead to chronic pain, difficulty walking, and postural imbalances. In young children, deep burns over growth plates may result in skeletal deformities and long-term orthopedic problems. During the acute phase of healing, a non-weight-bearing approach is recommended to protect the healing tissue and prevent wound breakdown. Once the initial healing is complete, custom orthotics and physiotherapy can help maintain proper foot alignment and restore normal function [107,108,109].

Genital and perineal burns, while less common, require specialized management due to the sensitivity of the tissues and their important functional roles. The area is highly vascularized, which promotes rapid healing; however, scarring and contractures can lead to complications such as urinary retention, strictures, phimosis, or vaginal stenosis, potentially affecting future reproductive and sexual function.

Urinary catheterization is often necessary to prevent urinary retention and reduce the risk of secondary infections. Bladder catheterization (with a Foley catheter) is important in severely burned patients to monitor the adequacy of fluid resuscitation [104].

In cases where scarring results in functional impairment, early surgical intervention may be required to avoid long-term complications. Additionally, considering the psychological impact of genital burns—especially in adolescents—it is important to integrate long-term monitoring and counseling into the care plan to support self-esteem and body image [102].

### 5.3. Nutritional Care

Nutritional care is a critical component in the management of pediatric burn patients. A hypermetabolic state typically follows the injury, and all efforts should be made to mitigate its consequences.

Burns result in a complex disease, with a high-demand metabolic state even higher compared to other critical states. The pediatric population is even more at risk since the catabolic state induces a dispense of the reserves that are limited in early-age patients [110,111].

Enteral nutrition via nasogastric tube is considered standard care for extensive burns, as it provides high-calorie, high-protein support, leading to significant improvements in the nutritional status of the patient [112]. The timing of starting enteral nutrition is very important, and early nutrition should be achieved in the pediatric population since it improves clinical outcomes, as shown by Shahi et al. [113]. Early enteral nutrition in burn patients has proven to be a safe and effective intervention, helping to reduce stress hormones and hypermetabolism, minimize catabolic processes, enhance immunoglobulin production, lower the incidence of stress ulcers, shorten intensive care unit (ICU) and hospital stays, and decrease mortality rates [110,113]. A delay in the initiation of enteral nutrition has been identified as a risk factor for poor outcomes and delayed recovery [113,114]. To overcome practical difficulties, many studies have been looking into intraoperative feeding, and there is strong evidence that intraoperative feeding is a safe and feasible option for providing enteral nutrition and ensuring adequate caloric intake [110,115,116].

Parenteral nutrition has been discussed as an alternative to enteral nutrition when not possible; maintaining a high intake of calories and nutrients is fundamental for the healing process and should not be interrupted. Burn-related inflammation leads to significant alterations in body composition, affecting both body mass and bone mineral density. However, an accurate assessment should be conducted to determine the specific needs of each patient throughout all phases of the healing process, including rehabilitation and follow-up care [117,118].

Burned patients require a tailored nutritional approach due to their increased metabolic demands. Specifically, protein needs are significantly increased in burn patients due to losses through urine and wounds, heightened demand for gluconeogenesis, and the process of wound healing. High-protein diets have shown positive outcomes, particularly in children, where protein intake (around 23% of total calories) enhances immune function, reduces bacteremia, and improves survival rates. As the burn size increases, greater protein intake is necessary to achieve a positive nitrogen balance, typically ranging from 1.5 to 3.0 g/kg/day [119,120,121].

Fats constitute a significant portion of caloric intake (30–50%) in critically ill patients to reduce carbohydrate needs and improve glucose tolerance. However, excessive fat intake can lead to complications such as hyperlipidemia, hepatic steatosis, hypoxemia, and increased infection risk. Guidelines recommend limiting fat intake to below 35% of total energy intake (TEI) [119].

Finally, carbohydrates should provide 55–60% of TEI but not exceed 5 mg/kg/min to prevent complications associated with excessive carbohydrate administration [122].

Concerning micronutrients, burn patients have higher requirements for vitamins C, A, and D and Zinc to support immune function and aid wound repair. Additionally, research suggests that nutritional supplementation with antioxidants such as vitamin E, vitamin C, and selenium may be useful to reduce oxidative damage. Studies indicate that antioxidant therapy may enhance immune function, support wound healing, and reduce the incidence of complications. However, the efficacy and safety of these interventions in pediatric burn patients remain uncertain, and further large-scale clinical trials are needed to establish evidence-based guidelines [10].

### 5.4. Pharmacological Modulation

Pharmacological modulation plays a pivotal role in the pediatric management of stress responses following burn trauma, addressing both physiological and psychological sequelae. As stated earlier, burn injuries in children induce a hypermetabolic state characterized by elevated levels of stress hormones and inflammatory cytokines, which can exacerbate tissue damage, impair wound healing, and hinder overall recovery. Recent improvements in understanding the hypermetabolic response to severe burns, along with advancements in critical care and infection management, have greatly reduced morbidity for burn survivors.

Pharmacological interventions aim to attenuate this maladaptive stress response while promoting an environment conducive to healing [2].

Agents such as beta-adrenergic blockers, exemplified by propranolol, are employed to mitigate hypermetabolism by reducing heart rate, energy expenditure, myocardial oxygen consumption, and protein catabolism [77]. This anti-catabolic therapy was shown to attenuate lipolysis, reduce hepatic fat accumulation, and improve insulin sensitivity [123]. Additionally, Hendron et al. found that beta-blockers, such as propranolol, have proven effective in reducing the hypermetabolic response by lowering catecholamine activity, which decreases protein breakdown and improves nitrogen balance [21].

Similar to propranolol, the exogenous administration of human GH (rHGH) by daily intramuscular injection significantly reduces the loss of lean body mass in patients but has mainly been studied in children, and combinations with propranolol have also shown benefit [124].

In addition, rhGH significantly reduced the serum levels of tumor necrosis factor-α, interleukin-1, serum amyloid A, and C-reactive protein and improved the hepatic acute-phase response, increased albumin synthesis, and demonstrated beneficial effects on weight gain, bone mineral content, growth velocity, and cardiac function, which persist in the long term.

GH therapy has been also investigated as a potential intervention to address the adverse effects of GH resistance. Research conducted by Suman and Herndon [16,30,125], indicates that administering recombinant GH can help maintain lean body mass, enhance protein synthesis, and accelerate wound healing in children with severe burns. Additionally, GH therapy has been associated with improved linear growth rates and better rehabilitation outcomes. For instance, Suman et al. [25,35] documented increases in muscle strength and enhanced exercise capacity in pediatric burn patients treated with GH. Nonetheless, concerns persist regarding the long-term safety and effectiveness of GH therapy in children. Possible side effects include hyperglycemia, fluid retention, and intracranial hypertension, which necessitate careful monitoring during treatment [17,25,35,126]. Given these challenges, further research is essential to optimize GH dosing protocols and identify pediatric burn patients who would most benefit from this treatment while minimizing associated risks.

Concurrently, Przkora et al. [127] support the administration of anabolic agents, including oxandrolone, an analog of testosterone. Their results showed that long-term oxandrolone treatment in severely burned pediatric patients effectively counters the persistent hypermetabolic state and promotes lean body mass accrual, bone mineral content and density, and muscle preservation. It also positively modulates anabolic and catabolic hormone profiles, increasing IGF-1 and testosterone levels and reducing cortisol. In recent years, there have been significant improvements in nitrogen retention and lean body mass in pediatric burn patients receiving oxandrolone [76,128,129]. These changes are associated with improved recovery outcomes, including better growth, wound healing, and overall physical rehabilitation.

Other anabolic agents still under investigation include metformin, IGF-1 combined with IGFBP-3, GLP-1, and PPAR-γ agonists [124]. Other therapeutic strategies, such as pamidronate or hormonal supplementation, may mitigate dysfunctional bone remodeling. A randomized double-blind controlled trial conducted by Borsheim and colleagues investigated the effects of a single dose of bisphosphonate administered within ten days following severe burn injury. The study revealed that this treatment not only suppressed muscle protein degradation but also enhanced muscle protein synthesis, resulting in a net anabolic effect compared to a placebo. Supporting this observation, the muscle fiber diameter was significantly larger in pamidronate-treated patients at 30 days post-burn, and at 9 months, peak lower limb torque in these patients was comparable to that of age- and sex-matched non-burned children. In contrast, placebo-treated patients showed a trend toward persistent lower limb weakness. These findings suggest that the pathophysiology of post-burn cachexia may involve factors released during bone resorption [15,130].

Glucose metabolism was similarly disrupted, with hyperglycemia and hepatic insulin resistance observed, underscoring the need for interventions targeting glucose regulation between 130 and 150 mg/dL. Insulin therapy has demonstrated considerable benefits for pediatric burn patients. Jeschke et al. [67] found that insulin administration reduces infection rates, fosters anabolic effects through enhanced protein synthesis, and helps preserve muscle mass, all of which are vital for functional recovery and growth. It promotes glucose uptake in muscle and adipose tissues, enhances protein synthesis, and inhibits proteolysis but carries a risk of hypoglycemia [16,30]. Metformin, instead, offers a safer alternative by improving glucose control. Recently, Rivas et al. [131] tested the hypothesis that combining metformin with a 6-week exercise program may further improve outcomes in burned children.

While thyroid hormone supplementation has also been proposed as a treatment option, its application in pediatric burn patients remains a topic of debate [132]. Additionally, further research specifically targeting pediatric populations is necessary to develop safe and effective treatment strategies [59].

Burn injuries frequently result in progressive vitamin D deficiency, primarily due to the compromised ability of injured and adjacent skin to convert 7-dehydrocholesterol into vitamin D upon ultraviolet B radiation exposure, compounded by insufficient vitamin D supplementation during or following acute burn care. Considering the established importance of vitamin D in maintaining muscle function, reestablishing normal circulating levels may contribute to the enhancement of bone and muscle health during the recovery phase post-burn [133]. Beyond metabolic stabilization, analgesics and sedatives are integral for managing pain and anxiety, which are substantial contributors to the neuroendocrine stress cascade.

Pardesi et al. [85] explored the challenges and advancements in treating pain in children who suffer from burn injuries. Nonsteroidal anti-inflammatory drugs are minimally studied in burn patients due to side-effect risks (gastrointestinal complications, bleeding risk, and renal toxicity). Ketamine is a well-established analgesic and sedative in pediatric burn care, particularly during painful procedures such as dressing changes and debridement, characterized by a favorable safety profile compared to opioids. Benzodiazepines, such as midazolam, are commonly used in pediatric burn patients to manage procedural anxiety and agitation. Medications often prescribed for managing neuropathic pain in children include tricyclic antidepressants, such as nortriptyline and amitriptyline, along with anticonvulsants like gabapentin and pregabalin. Alongside conventional pharmacological treatments for managing background, procedural, and perioperative pain, increasing evidence supports the use of regional anesthesia and innovative technologies like virtual reality. However, numerous clinical questions remain unanswered in the treatment of burn patients, highlighting the need for further research in various areas to enhance care for these children.

Moreover, anti-inflammatory medications, including corticosteroids and cytokine inhibitors, are being explored to suppress the overactive inflammatory response. Cortisol-blocking strategies, including ketoconazole, are under investigation [16,134].

## 6. Limitations

Certainly, this review has its limitations. To begin with, it is presented as a narrative review, which, as highlighted by Gregory et al. [135], provides a non-systematic summary and evaluation of the available literature on a specific subject. The lack of a systematic approach in narrative reviews implies the absence of rigorously defined methodologies, which can lead to selection biases and predominantly qualitative analyses. For example, this review exclusively considers articles retrieved from PubMed and the Web of Science, including manuscripts published only in English, potentially overlooking relevant studies accessible through other databases or search platforms. The literature review highlights that high-quality clinical studies on pediatric burn patients are often scarce. Most of the existing research is observational or based on small sample sizes, which reduces the robustness of the conclusions. Data heterogeneity is another significant issue, as studies often involve diverse pediatric populations with varying degrees of burn severity, age ranges, and comorbidities, complicating direct comparisons. Furthermore, due to the paucity of pediatric-focused studies, this review may have incorporated data from adult populations, which can limit the applicability of the conclusions. Additionally, age-specific variations and developmental changes in hormonal and metabolic regulation pose challenges in standardizing results across different age groups. Variability in diagnostic criteria and methods for measuring hormonal and metabolic changes can further hinder meaningful comparisons between studies. Similarly, the diversity of therapeutic approaches, coupled with the absence of standardized protocols, often leads to inconsistencies in the reported outcomes, emphasizing the need for large-scale clinical studies. Finally, we focused on the complex response occurring in major burns; however, changes can also occur in pediatric burns of varying degrees. Studies comparing different responses are mandatory for a better understanding of the physiological response to burns.

## 7. Conclusions

Pediatric burn injuries trigger profound metabolic and physiological responses that, if not properly managed, can lead to long-term morbidity. Effective management of the physiological stress response in pediatric burn patients necessitates a dedicated multidisciplinary approach that integrates medical, nutritional, surgical, and rehabilitative strategies. Initiating early enteral nutrition is a cornerstone of burn care for children, as it helps to counteract hypermetabolism and catabolic stress. Timely nutritional support preserves muscle mass, promotes wound healing, and reduces the risk of complications, including stress ulcers, infections, and organ dysfunction. An individualized nutritional plan that emphasizes the optimal intake of macronutrients (protein, fat, and carbohydrates) alongside targeted supplementation of micronutrients and antioxidants is essential to achieving positive clinical outcomes. Advances in pharmacological interventions, such as beta-blockers, anabolic agents, and hormonal treatment, offer promising pathways to improve recovery and mitigate long-term complications. Early mobilization and physiotherapy are vital in mitigating the adverse effects of prolonged immobility, such as muscle wasting, joint contractures, and functional decline, and are strictly related to the advancement devices and techniques that promote the use of less invasive procedures, regenerative medicine, and reconstructive technique especially in small patients. While current strategies have significantly improved the survival and outcomes of pediatric burn patients, ongoing research is critical to refine these approaches. Large-scale clinical trials are needed to evaluate the safety and efficacy of emerging therapies, such as beta-blockers and anabolic agents, in diverse pediatric populations. Additionally, the early management of the debridement phase must be tailored to pediatric patients, including enzymatic debridement with bromelain-based products [93]. The use of new technologies to minimize pain and anxiety during repeated procedures should also be considered [81]. Finally, the long-term impact of nutritional and rehabilitative interventions on growth, development, and quality of life remains an area of active investigation.

## Figures and Tables

**Table 1:** Key scientific studies on burn patients, outlining the main findings and characteristics of the research.

Author(s)	Population Studied	Result	Conclusions
Williams FN et al., 2009 [23]	Mixed-age burn patients with severe injuries	The hypermetabolic response increases resting energy expenditure (REE), protein catabolism, and delays wound healing. Targeted therapies like beta-blockers and insulin showed improvements.	Early management of the hypermetabolic response improves survival, reduces complications, and enhances recovery.
Jeschke MG, et al., 2009 [22]	Burn patients with hepatic complications	The liver plays a critical role in the hypermetabolic response through altered gluconeogenesis, inflammation, and acute phase protein synthesis.	Optimizing hepatic function through tailored pharmacological and nutritional interventions significantly impacts patient outcomes.
Jeschke MG, et al., 2008 [17]	Severely burned adults and children	Burns trigger systemic responses, including inflammation, insulin resistance, and increased REE, with immune system dysfunction prolonging recovery.	Understanding pathophysiology enables development of therapies targeting metabolic, immune, and inflammatory responses.
Porter C, et al., 2016 [37]	Burn patients with focus on metabolic recovery	Burn trauma induces significant metabolic stress, exacerbated by inflammation and insulin resistance, impacting muscle and bone integrity.	Emerging therapies, including pharmacological and nutritional approaches, are essential to modulate metabolic stress and improve outcomes.
Jeschke MG, et al., 2011 [4]	Burn patients with systemic metabolic dysfunction	Pathophysiologic responses persist long-term, including inflammation, insulin resistance, and muscle atrophy.	Long-term monitoring and integrated care plans are critical for addressing systemic effects in survivors.
Jeschke MG, et al., 2007 [76]	Pediatric burn patients with >40% total body surface area (TBSA) burns	Larger burns correlate with more severe metabolic and inflammatory responses, including elevated cytokines and catecholamines.	Tailoring therapy based on TBSA improves survival rates and recovery outcomes in pediatric patients.
Williams FN, et al., 2017 [16]	Pediatric burn patients with long-term follow-up	Long-term hypermetabolic stress impacts growth parameters, including delayed linear growth, muscle mass depletion, and reduced bone density.	Long-term care involving metabolic and nutritional interventions is necessary to mitigate growth and developmental delays.
Suman OE, et al., 2006 [25]	Severely burned children engaged in exercise trials	Exercise revealed significant deficits in aerobic and anaerobic capacity, exacerbating physical recovery challenges.	Structured rehabilitation programs are essential to rebuild energy systems and optimize functional outcomes in severely burned children.
Jeschke MG, et al., 2020 [1]	Pediatric burn survivors across multiple studies	Longitudinal studies reveal persistent growth impairments, insulin resistance, and ongoing metabolic challenges in burn survivors.	A multidisciplinary approach involving endocrinologists, dieticians, and physical therapists is necessary for long-term recovery and quality of life.
Porter C, et al., 2016 [34]	Pediatric burn survivors undergoing rehabilitation	Immobilization worsens muscle and bone loss; early rehabilitation preserves skeletal health, reduces muscle catabolism, and enhances functional outcomes.	Rehabilitation must be integral to post-burn care to counteract immobilization’s detrimental effects and promote recovery.
Clark A, et al., 2017 [10]	Burn patients undergoing nutritional assessment	High-protein diets and calorie optimization showed significant improvements in muscle preservation and wound healing.	Tailored nutritional strategies enhance outcomes, reduce infections, and shorten recovery time, emphasizing individualized care.
Jeschke MG, et al., 2004 [28]	Pediatric burn patients with severe burns	Severe burns lead to prolonged hypermetabolism, affecting hepatic protein production and immune function.	Long-term metabolic monitoring is critical in children with severe burns to address hepatic dysfunction and systemic effects.
Jeschke MG, et al., 2007 [77]	Pediatric burn patients with propranolol treatment	Propranolol reduces heart rate, REE, and muscle breakdown without increasing inflammation or infection risks.	Propranolol is effective and safe in reducing hypermetabolism and preserving muscle mass in pediatric burn patients.

## Data Availability

Not applicable.

## References

[1] Jeschke M.G., Van Baar M.E., Choudhry M.A., Chung K.K., Gibran N.S., Logsetty S. (2020). Burn Injury. Nat. Rev. Dis. Primer.

[2] Partain K.P., Fabia R., Thakkar R.K. (2020). Pediatric Burn Care: New Techniques and Outcomes. Curr. Opin. Pediatr..

[3] Gonzalez R., Shanti C.M. (2015). Overview of Current Pediatric Burn Care. Semin. Pediatr. Surg..

[4] Jeschke M.G., Gauglitz G.G., Kulp G.A., Finnerty C.C., Williams F.N., Kraft R., Suman O.E., Mlcak R.P., Herndon D.N. (2011). Long-Term Persistance of the Pathophysiologic Response to Severe Burn Injury. PLoS ONE.

[5] Sommerhalder C., Blears E., Murton A.J., Porter C., Finnerty C., Herndon D.N. (2020). Current Problems in Burn Hypermetabolism. Curr. Probl. Surg..

[6] Arbuthnot M.K., Garcia A.V. (2019). Early Resuscitation and Management of Severe Pediatric Burns. Semin. Pediatr. Surg..

[7] Berry J., Stone K., Reid J., Bell A., Burns R. (2018). Pediatric Emergency Medicine Simulation Curriculum: Electrical Injury. MedEdPORTAL.

[8] Jeschke M.G., Herndon D.N. (2014). Burns in Children: Standard and New Treatments. Lancet.

[9] Fenlon S., Siddarth N. (2007). Burns in Children. BJA Educ..

[10] Clark A., Imran J., Madni T., Wolf S.E. (2017). Nutrition and Metabolism in Burn Patients. Burns Trauma.

[11] Abboud E.C., Legare T.B., Settle J.C., Boubekri A.M., Barillo D.J., Marcet J.E., Sanchez J.E. (2014). Do Silver-Based Wound Dressings Reduce Pain? A Prospective Study and Review of the Literature. Burns.

[12] Reed J.L., Pomerantz W.J. (2005). Emergency Management of Pediatric Burns. Pediatr. Emerg. Care.

[13] Wang Y., Beekman J., Hew J., Jackson S., Issler-Fisher A.C., Parungao R., Lajevardi S.S., Li Z., Maitz P.K.M. (2018). Burn Injury: Challenges and Advances in Burn Wound Healing, Infection, Pain and Scarring. Adv. Drug Deliv. Rev..

[14] Toma A., Voicu D., Popazu C., Mihalache D., Duca O., Dănilă D.M., Enescu D.M. (2024). Severity and Clinical Outcomes of Pediatric Burns—A Comprehensive Analysis of Influencing Factors. J. Pers. Med..

[15] Klein G.L. (2019). The Role of the Musculoskeletal System in Post-Burn Hypermetabolism. Metabolism.

[16] Williams F.N., Herndon D.N. (2017). Metabolic and Endocrine Considerations After Burn Injury. Clin. Plast. Surg..

[17] Jeschke M.G., Chinkes D.L., Finnerty C.C., Kulp G., Suman O.E., Norbury W.B., Branski L.K., Gauglitz G.G., Mlcak R.P., Herndon D.N. (2008). Pathophysiologic Response to Severe Burn Injury. Ann. Surg..

[18] Korzeniowski T., Mertowska P., Mertowski S., Podgajna M., Grywalska E., Strużyna J., Torres K. (2022). The Role of the Immune System in Pediatric Burns: A Systematic Review. J. Clin. Med..

[19] Herndon D.N. (2018). Total Burn Care.

[20] Guillory A.N., Porter C., Suman O.E., Zapata-Sirvent R.L., Finnerty C.C., Herndon D.N. (2018). Modulation of the Hypermetabolic Response After Burn Injury. Total Burn Care.

[21] Herndon D.N., Hart D.W., Wolf S.E., Chinkes D.L., Wolfe R.R. (2001). Reversal of Catabolism by Beta-Blockade After Severe Burns. N. Engl. J. Med..

[22] Jeschke M.G. (2009). The Hepatic Response to Thermal Injury: Is the Liver Important for Postburn Outcomes?. Mol. Med..

[23] Williams F.N., Herndon D.N., Jeschke M.G. (2009). The Hypermetabolic Response to Burn Injury and Interventions to Modify This Response. Clin. Plast. Surg..

[24] Jeschke M.G. (2016). Postburn Hypermetabolism: Past, Present, and Future. J. Burn Care Res..

[25] Suman O.E., Mlcak R.P., Chinkes D.L., Herndon D.N. (2006). Resting Energy Expenditure in Severely Burned Children: Analysis of Agreement Between Indirect Calorimetry and Prediction Equations Using the Bland–Altman Method. Burns.

[26] Yeolekar L.R., Damle R.G., Kamat A.N., Khude M.R., Simha V., Pandit A.N. (2008). Respiratory Viruses in Acute Respiratory Tract Infections in Western India. Indian J. Pediatr..

[27] Toliver-Kinsky T., Kobayashi M., Suzuki F., Sherwood E.R. (2018). The Systemic Inflammatory Response Syndrome. Total Burn Care.

[28] Jeschke M.G. (2004). Extended Hypermetabolic Response of the Liver in Severely Burned Pediatric Patients. Arch. Surg..

[29] Dotan R., Mitchell C., Cohen R., Klentrou P., Gabriel D., Falk B. (2012). Child—Adult Differences in Muscle Activation—A Review. Pediatr. Exerc. Sci..

[30] Knuth C.M., Auger C., Jeschke M.G. (2021). Burn-Induced Hypermetabolism and Skeletal Muscle Dysfunction. Am. J. Physiol.-Cell Physiol..

[31] Klein G.L. (2018). The Role of Calcium in Inflammation-Associated Bone Resorption. Biomolecules.

[32] Hart D.W., Wolf S.E., Chinkes D.L., Gore D.C., Mlcak R.P., Beauford R.B., Obeng M.K., Lal S., Gold W.F., Wolfe R.R. (2000). Determinants of Skeletal Muscle Catabolism After Severe Burn. Ann. Surg..

[33] Schryver E., Klein G.L., Herndon D.N., Suman O.E., Branski L.K., Sousse L.E. (2018). Bone Metabolism in Pediatric Burned Patients: A Review. Burns.

[34] Porter C., Herndon D.N., Børsheim E., Bhattarai N., Chao T., Reidy P.T., Rasmussen B.B., Andersen C.R., Suman O.E., Sidossis L.S. (2016). Long-Term Skeletal Muscle Mitochondrial Dysfunction Is Associated with Hypermetabolism in Severely Burned Children. J. Burn Care Res..

[35] Suman O.E., Spies R.J., Celis M.M., Mlcak R.P., Herndon D.N. (2001). Effects of a 12-Wk Resistance Exercise Program on Skeletal Muscle Strength in Children with Burn Injuries. J. Appl. Physiol..

[36] Hart D.W., Wolf S.E., Mlcak R., Chinkes D.L., Ramzy P.I., Obeng M.K., Ferrando A.A., Wolfe R.R., Herndon D.N. (2000). Persistence of Muscle Catabolism After Severe Burn. Surgery.

[37] Porter C., Tompkins R.G., Finnerty C.C., Sidossis L.S., Suman O.E., Herndon D.N. (2016). The Metabolic Stress Response to Burn Trauma: Current Understanding and Therapies. Lancet.

[38] Cree M.G., Wolfe R.R. (2008). Postburn Trauma Insulin Resistance and Fat Metabolism. Am. J. Physiol.-Endocrinol. Metab..

[39] Abdullahi A., Samadi O., Auger C., Kanagalingam T., Boehning D., Bi S., Jeschke M.G. (2019). Browning of White Adipose Tissue After a Burn Injury Promotes Hepatic Steatosis and Dysfunction. Cell Death Dis..

[40] Johnson B.Z., McAlister S., McGuire H.M., Palanivelu V., Stevenson A., Richmond P., Palmer D.J., Metcalfe J., Prescott S.L., Wood F.M. (2020). Pediatric Burn Survivors Have Long-Term Immune Dysfunction with Diminished Vaccine Response. Front. Immunol..

[41] Ogunbileje J.O., Herndon D.N., Murton A.J., Porter C. (2017). The Role of Mitochondrial Stress in Muscle Wasting Following Severe Burn Trauma. J. Burn Care Res..

[42] Clayton R.P., Herndon D.N., Abate N., Porter C. (2018). The Effect of Burn Trauma on Lipid and Glucose Metabolism: Implications for Insulin Sensitivity. J. Burn Care Res..

[43] Jeschke M.G., Norbury W.B., Finnerty C.C., Mlcak R.P., Kulp G.A., Branski L.K., Gauglitz G.G., Herndon B., Swick A., Herndon D.N. (2008). Age Differences in Inflammatory and Hypermetabolic Postburn Responses. Pediatrics.

[44] Shields B.A., VanFosson C.A., Pruskowski K.A., Gurney J.M., Rizzo J.A., Cancio L.C. (2019). High-Carbohydrate vs High-Fat Nutrition for Burn Patients. Nutr. Clin. Pract..

[45] Alloju S.M., Herndon D.N., McEntire S.J., Suman O.E. (2008). Assessment of Muscle Function in Severely Burned Children. Burns.

[46] Kulp G.A., Herndon D.N., Lee J.O., Suman O.E., Jeschke M.G. (2010). Extent and magnitude of catecholamine surge in pediatric burned patients. Shock.

[47] Dombrecht D., Van Daele U., Van Asbroeck B., Schieffelers D., Guns P., Gebruers N., Meirte J., Van Breda E. (2023). Molecular Mechanisms of Post-Burn Muscle Wasting and the Therapeutic Potential of Physical Exercise. J. Cachexia Sarcopenia Muscle.

[48] Ballard-Croft C., Maass D.L., Sikes P., White J., Horton J. (2002). Activation of Stress-Responsive Pathways by the Sympathetic Nervous System in Burn Trauma. Shock.

[49] Mecott G.A., Al-Mousawi A.M., Gauglitz G.G., Herndon D.N., Jeschke M.G. (2010). The role of hyperglycemia in burned patients: Evidence-based studies. Shock.

[50] Norbury W.B., Herndon D.N., Branski L.K., Chinkes D.L., Jeschke M.G. (2008). Urinary Cortisol and Catecholamine Excretion After Burn Injury in Children. J. Clin. Endocrinol. Metab..

[51] Greenhalgh D.G. (2017). Sepsis in the Burn Patient: A Different Problem than Sepsis in the General Population. Burns Trauma.

[52] Auger C., Samadi O., Jeschke M.G. (2017). The Biochemical Alterations Underlying Post-Burn Hypermetabolism. Biochim. Biophys. Acta BBA-Mol. Basis Dis..

[53] Pin F., Bonetto A., Bonewald L.F., Klein G.L. (2019). Molecular Mechanisms Responsible for the Rescue Effects of Pamidronate on Muscle Atrophy in Pediatric Burn Patients. Front. Endocrinol..

[54] Finnerty C.C., Jeschke M.G., Branski L.K., Barret J.P., Dziewulski P., Herndon D.N. (2016). Hypertrophic Scarring: The Greatest Unmet Challenge After Burn Injury. Lancet.

[55] Mathis S.L., Farley R.S., Fuller D.K., Jetton A.E., Caputo J.L. (2013). The Relationship between Cortisol and Bone Mineral Density in Competitive Male Cyclists. J. Sports Med..

[56] Pérez-Guisado J., De Haro-Padilla J.M., Rioja L.F., DeRosier L.C., De La Torre J.I. (2013). The Potential Association of Later Initiation of Oral/Enteral Nutrition on Euthyroid Sick Syndrome in Burn Patients. Int. J. Endocrinol..

[57] McIVER B., Gorman C.A. (1997). Euthyroid Sick Syndrome: An Overview. Thyroid.

[58] Radman M., Portman M. (2016). Thyroid Hormone in the Pediatric Intensive Care Unit. J. Pediatr. Intensive Care.

[59] Barclay C., Sedowofia K., Thomson M., McIntosh N. (1996). Thyroid hormones in burn injured children. Biochem. Soc. Trans..

[60] Safer J.D. (2013). Thyroid Hormone and Wound Healing. J. Thyroid Res..

[61] Mitchell C.S., Savage D.B., Dufour S., Schoenmakers N., Murgatroyd P., Befroy D., Halsall D., Northcott S., Raymond-Barker P., Curran S. (2010). Resistance to Thyroid Hormone Is Associated with Raised Energy Expenditure, Muscle Mitochondrial Uncoupling, and Hyperphagia. J. Clin. Investig..

[62] Mathias E., Srinivas Murthy M. (2017). Pediatric Thermal Burns and Treatment: A Review of Progress and Future Prospects. Medicines.

[63] Suman A., Owen J. (2020). Update on the Management of Burns in Paediatrics. BJA Educ..

[64] Mullur R., Liu Y.-Y., Brent G.A. (2014). Thyroid Hormone Regulation of Metabolism. Physiol. Rev..

[65] Przkora R., Barrow R.E., Jeschke M.G., Suman O.E., Celis M., Sanford A.P., Chinkes D.L., Mlcak R.P., Herndon D.N. (2006). Body Composition Changes with Time in Pediatric Burn Patients. J. Trauma Inj. Infect. Crit. Care.

[66] Finnerty C.C., Ali A., McLean J., Benjamin N., Clayton R.P., Andersen C.R., Mlcak R.P., Suman O.E., Meyer W., Herndon D.N. (2014). Impact of Stress-Induced Diabetes on Outcomes in Severely Burned Children. J. Am. Coll. Surg..

[67] Jeschke M.G., Kulp G.A., Kraft R., Finnerty C.C., Mlcak R., Lee J.O., Herndon D.N. (2010). Intensive Insulin Therapy in Severely Burned Pediatric Patients: A Prospective Randomized Trial. Am. J. Respir. Crit. Care Med..

[68] Chondronikola M., Meyer W.J., Sidossis L.S., Ojeda S., Huddleston J., Stevens P., Børsheim E., Suman O.E., Finnerty C.C., Herndon D.N. (2014). Predictors of Insulin Resistance in Pediatric Burn Injury Survivors 24 to 36 Months Postburn. J. Burn Care Res..

[69] Gauglitz G.G., Herndon D.N., Kulp G.A., Meyer W.J., Jeschke M.G. (2009). Abnormal Insulin Sensitivity Persists up to Three Years in Pediatric Patients Post-Burn. J. Clin. Endocrinol. Metab..

[70] Herndon D.N. (1994). Lipolysis in Burned Patients Is Stimulated by the Β2-Receptor for Catecholamines. Arch. Surg..

[71] Herndon D.N., Hawkins H.K., Nguyen T.T., Pierre E., Cox R., Barrow R.E. (1995). Characterization of Growth Hormone Enhanced Donor Site Healing in Patients with Large Cutaneous Burns. Ann. Surg..

[72] Rojas Y., Finnerty C.C., Radhakrishnan R.S., Herndon D.N. (2012). Burns: An Update on Current Pharmacotherapy. Expert Opin. Pharmacother..

[73] Diaz E.C., Herndon D.N., Lee J., Porter C., Cotter M., Suman O.E., Sidossis L.S., Børsheim E. (2015). Predictors of Muscle Protein Synthesis After Severe Pediatric Burns. J. Trauma Acute Care Surg..

[74] Patel K.F., Rodríguez-Mercedes S.L., Grant G.G., Rencken C.A., Kinney E.M., Austen A., Hou C., Brady K.J.S., Schneider J.C., Kazis L.E. (2022). Physical, Psychological, and Social Outcomes in Pediatric Burn Survivors Ages 5 to 18 Years: A Systematic Review. J. Burn Care Res..

[75] Szabo M.M., Ferris K.A., Urso L., Aballay A.M., Duncan C.L. (2017). Social Competence in Pediatric Burn Survivors: A Systematic Review. Rehabil. Psychol..

[76] Jeschke M.G., Mlcak R.P., Finnerty C.C., Norbury W.B., Gauglitz G.G., Kulp G.A., Herndon D.N. (2007). Burn Size Determines the Inflammatory and Hypermetabolic Response. Crit. Care.

[77] Jeschke M.G., Norbury W.B., Finnerty C.C., Branski L.K., Herndon D.N. (2007). Propranolol Does Not Increase Inflammation, Sepsis, or Infectious Episodes in Severely Burned Children. J. Trauma Inj. Infect. Crit. Care.

[78] Storey K., Kimble R.M., Holbert M.D. (2021). The Management of Burn Pain in a Pediatric Burns-Specialist Hospital. Pediatr. Drugs.

[79] Brown N.J., Kimble R.M., Rodger S., Ware R.S., Cuttle L. (2014). Play and Heal: Randomized Controlled Trial of Ditto^TM^ Intervention Efficacy on Improving Re-Epithelialization in Pediatric Burns. Burns.

[80] Chester S.J., Stockton K., De Young A., Kipping B., Tyack Z., Griffin B., Chester R.L., Kimble R.M. (2016). Effectiveness of Medical Hypnosis for Pain Reduction and Faster Wound Healing in Pediatric Acute Burn Injury: Study Protocol for a Randomized Controlled Trial. Trials.

[81] Chester S.J., Tyack Z., De Young A., Kipping B., Griffin B., Stockton K., Ware R.S., Zhang X., Kimble R.M. (2018). Efficacy of Hypnosis on Pain, Wound-Healing, Anxiety, and Stress in Children with Acute Burn Injuries: A Randomized Controlled Trial. Pain.

[82] Boccella S., De Filippis L., Giorgio C., Brandolini L., Jones M., Novelli R., Amorizzo E., Leoni M.L.G., Terranova G., Maione S. (2023). Combination Drug Therapy for the Management of Chronic Neuropathic Pain. Biomolecules.

[83] Ratcliff S.L., Brown A., Rosenberg L., Rosenberg M., Robert R.S., Cuervo L.J., Villarreal C., Thomas C.R., Meyer W.J. (2006). The Effectiveness of a Pain and Anxiety Protocol to Treat the Acute Pediatric Burn Patient. Burns.

[84] Cox S.G., Martinez R., Glick A., Numanoglu A., Rode H. (2015). A Review of Community Management of Paediatric Burns. Burns.

[85] Pardesi O., Fuzaylov G. (2017). Pain Management in Pediatric Burn Patients: Review of Recent Literature and Future Directions. J. Burn Care Res..

[86] Sharma R., Parashar A. (2010). Special Considerations in Paediatric Burn Patients. Indian J. Plast. Surg..

[87] Brown M., Coffee T., Adenuga P., Yowler C.J. (2014). Outcomes of Outpatient Management of Pediatric Burns. J. Burn Care Res..

[88] Xiao-Wu W. (2002). Effects of Delayed Wound Excision and Grafting in Severely Burned Children. Arch. Surg..

[89] Pietsch J.B., Netscher D.T., Nagaraj H.S., Groff D.B. (1985). Early Excision of Major Burns in Children: Effect on Morbidity and Mortality. J. Pediatr. Surg..

[90] Housinger T.A., Hills J., Warden G.D. (1994). Management of Pediatric Facial Burns. J. Burn Care Rehabil..

[91] Hirche C., Citterio A., Hoeksema H., Koller J., Lehner M., Martinez J.R., Monstrey S., Murray A., Plock J.A., Sander F. (2017). Eschar Removal by Bromelain Based Enzymatic Debridement (Nexobrid^®^) in Burns: An European Consensus. Burns.

[92] De Decker I., De Graeve L., Hoeksema H., Monstrey S., Verbelen J., De Coninck P., Vanlerberghe E., Claes K.E.Y. (2022). Enzymatic Debridement: Past, Present, and Future. Acta Chir. Belg..

[93] Shoham Y., Krieger Y., Rubin G., Koenigs I., Hartmann B., Sander F., Schulz A., David K., Rosenberg L., Silberstein E. (2020). Rapid Enzymatic Burn Debridement: A Review of the Paediatric Clinical Trial Experience. Int. Wound J..

[94] Korzeniowski T., Grywalska E., Strużyna J., Bugaj-Tobiasz M., Surowiecka A., Korona-Głowniak I., Staśkiewicz M., Torres K. (2022). Preliminary Single-Center Experience of Bromelain-Based Eschar Removal in Children with Mixed Deep Dermal and Full Thickness Burns. J. Clin. Med..

[95] Hart D.W., Wolf S.E., Beauford R.B., Lal S.O., Chinkes D.L., Herndon D.N. (2001). Determinants of Blood Loss during Primary Burn Excision. Surgery.

[96] Barret J.P. (2003). Modulation of Inflammatory and Catabolic Responses in Severely Burned Children by Early Burn Wound Excision in the First 24 Hours. Arch. Surg..

[97] Gupta S., Mohapatra D., Chittoria R., Subbarayan E., Reddy S., Chavan V., Aggarwal A., Reddy L. (2019). Human Skin Allograft: Is It a Viable Option in Management of Burn Patients?. J. Cutan. Aesthetic Surg..

[98] Branski L.K., Herndon D.N., Pereira C., Mlcak R.P., Celis M.M., Lee J.O., Sanford A.P., Norbury W.B., Zhang X.-J., Jeschke M.G. (2007). Longitudinal Assessment of Integra in Primary Burn Management: A Randomized Pediatric Clinical Trial. Crit. Care Med..

[99] Barret J.P., Dziewulski P., Wolf S.E., Desai M.H., Herndon D.N. (1999). Outcome of Scalp Donor Sites in 450 Consecutive Pediatric Burn Patients. Plast. Reconstr. Surg..

[100] Delgado-Miguel C., García Morán A., Fuentes Gómez L., Díaz M., Miguel-Ferrero M., López-Gutiérrez J.C. (2024). Comparison of the Effectiveness of Three Different Skin Substitutes for the Treatment of Pediatric Burns. Eur. J. Pediatr..

[101] Pedrazzi N.E., Naiken S., La Scala G. (2021). Negative Pressure Wound Therapy in Pediatric Burn Patients: A Systematic Review. Adv. Wound Care.

[102] Krishnamoorthy V., Ramaiah R., Bhananker S. (2012). Pediatric Burn Injuries. Int. J. Crit. Illn. Inj. Sci..

[103] Grande C.M., Stene J.K., Bernhard W.N. (1990). Airway Management: Considerations in the Trauma Patient. Crit. Care Clin..

[104] Sheridan R.L. (2002). Burns. Crit. Care Med..

[105] Fabia R., Groner J.I. (2009). Advances in the Care of Children with Burns. Adv. Pediatr..

[106] Sheridan R.L. (2000). Evaluating and Managing Burn Wounds. Dermatol. Nurs..

[107] Ngu F., Patel B., McBride C. (2017). Epidemiology of Isolated Foot Burns in Children Presenting to a Queensland Paediatric Burns Centre—A Two-Year Study in Warmer Climate. Burns Trauma.

[108] Waymack J.P., Fidler J., Warden G.D. (1988). Surgical Correction of Burn Scar Contractures of the Foot in Children. Burns.

[109] Singh K., Prasanna M. (1995). Tangential Excision and Skin Grafting for Ash Burns of the Foot in Children: A Preliminary Report. J. Trauma Inj. Infect. Crit. Care.

[110] Hudson A.S., Morzycki A.D., Wong J. (2022). Safety and Benefits of Intraoperative Enteral Nutrition in Critically Ill Pediatric Burn Patients: A Systematic Review and Pooled Analysis. J. Burn Care Res..

[111] Galfo M., De Bellis A., Melini F. (2018). Nutritional Therapy for Burns in Children. J. Emerg. Crit. Care Med..

[112] Endorf F.W., Ahrenholz D. (2011). Burn Management. Curr. Opin. Crit. Care.

[113] Shahi N., Skillman H.E., Phillips R., Cooper E.H., Shirek G.P., Goldsmith A., Meier M.R., Kaizer A.M., Recicar J.F., Banks A. (2021). Why Delay? Early Enteral Nutrition in Pediatric Burn Patients Improves Outcomes. J. Burn Care Res..

[114] Valentini M., Seganfredo F.B., Fernandes S.A. (2019). Pediatric Enteral Nutrition Therapy for Burn Victims: When Should It Be Initiated?. Rev. Bras. Ter. Intensiv..

[115] Sunderman C.A., Gottschlich M.M., Allgeier C., Warden G. (2019). Safety and Tolerance of Intraoperative Enteral Nutrition Support in Pediatric Burn Patients. Nutr. Clin. Pract..

[116] Imeokparia F., Johnson M., Thakkar R.K., Giles S., Capello T., Fabia R. (2018). Safety and Efficacy of Uninterrupted Perioperative Enteral Feeding in Pediatric Burn Patients. Burns.

[117] Prelack K., Yu Y.M., Sheridan R.L. (2015). Nutrition and Metabolism in the Rehabilitative Phase of Recovery in Burn Children: A Review of Clinical and Research Findings in a Speciality Pediatric Burn Hospital. Burns Trauma.

[118] Dylewksi M.L., Baker M., Prelack K., Weber J.M., Hursey D., Lydon M., Fagan S.P., Sheridan R.L. (2013). The Safety and Efficacy of Parenteral Nutrition Among Pediatric Patients with Burn Injuries. Pediatr. Crit. Care Med..

[119] Lee J.O., Gauglitz G.G., Herndon D.N., Hawkins H.K., Halder S.C., Jeschke M.G. (2011). Association Between Dietary Fat Content and Outcomes in Pediatric Burn Patients. J. Surg. Res..

[120] Chan M.M., Chan G.M. (2009). Nutritional Therapy for Burns in Children and Adults. Nutrition.

[121] Waymack J.P., Herndon D.N. (1992). Nutritional Support of the Burned Patient. World J. Surg..

[122] Rousseau A.-F., Losser M.-R., Ichai C., Berger M.M. (2013). ESPEN Endorsed Recommendations: Nutritional Therapy in Major Burns. Clin. Nutr..

[123] (2013). The Utility of Long-Term Propranolol in Pediatric Burn Patients. AAP Grand Rounds.

[124] Miguel Ferrero M., Díaz González M. (2022). Advances in the Treatment of Burned Children. Cir. Pediátrica.

[125] Mlcak R.P., Suman O.E., Murphy K., Herndon D.N. (2005). Effects of Growth Hormone on Anthropometric Measurements and Cardiac Function in Children with Thermal Injury. Burns.

[126] Herndon D.N., Ramzy P.I., DebRoy M.A., Zheng M., Ferrando A.A., Chinkes D.L., Barret J.P., Wolfe R.R., Wolf S.E. (1999). Muscle Protein Catabolism After Severe Burn: Effects of IGF-1/IGFBP-3 Treatment. Ann. Surg..

[127] Przkora R., Jeschke M.G., Barrow R.E., Suman O.E., Meyer W.J., Finnerty C.C., Sanford A.P., Lee J., Chinkes D.L., Mlcak R.P. (2005). Metabolic and Hormonal Changes of Severely Burned Children Receiving Long-Term Oxandrolone Treatment. Ann. Surg..

[128] Jeschke M.G., Finnerty C.C., Suman O.E., Kulp G., Mlcak R.P., Herndon D.N. (2007). The Effect of Oxandrolone on the Endocrinologic, Inflammatory, and Hypermetabolic Responses During the Acute Phase Postburn. Ann. Surg..

[129] Porro L.J., Herndon D.N., Rodriguez N.A., Jennings K., Klein G.L., Mlcak R.P., Meyer W.J., Lee J.O., Suman O.E., Finnerty C.C. (2012). Five-Year Outcomes after Oxandrolone Administration in Severely Burned Children: A Randomized Clinical Trial of Safety and Efficacy. J. Am. Coll. Surg..

[130] Børsheim E., Herndon D.N., Hawkins H.K., Suman O.E., Cotter M., Klein G.L. (2014). Pamidronate Attenuates Muscle Loss After Pediatric Burn Injury. J. Bone Miner. Res..

[131] Rivas E., Herndon D.N., Porter C., Meyer W., Suman O.E. (2018). Short-Term Metformin and Exercise Training Effects on Strength, Aerobic Capacity, Glycemic Control, and Mitochondrial Function in Children with Burn Injury. Am. J. Physiol.-Endocrinol. Metab..

[132] Sen S., Palmieri T., Greenhalgh D. (2018). Thyroid Storm in a Pediatric High Voltage Electrical Burn Injury. Burns Open.

[133] Gottschlich M.M., Mayes T., Khoury J., Kagan R.J. (2017). Clinical Trial of Vitamin D_2_ vs D_3_ Supplementation in Critically Ill Pediatric Burn Patients. J. Parenter. Enter. Nutr..

[134] Jeschke M.G., Williams F.N., Finnerty C.C., Rodriguez N.A., Kulp G.A., Ferrando A., Norbury W.B., Suman O.E., Kraft R., Branski L.K. (2012). The Effect of Ketoconazole on Post-Burn Inflammation, Hypermetabolism and Clinical Outcomes. PLoS ONE.

[135] Gregory A.T., Denniss A.R. (2018). An Introduction to Writing Narrative and Systematic Reviews—Tasks, Tips and Traps for Aspiring Authors. Heart Lung Circ..

